# La grossesse hétérotopique spontanée: à propos de deux cas: Spontaneous heterotopic pregnancy: about 2 cases

**DOI:** 10.11604/pamj.2017.28.306.13696

**Published:** 2017-12-12

**Authors:** Ahmed Guennoun, Nisrine Mamouni, Sanaa Errarhay, Chahrazad Bouchikhi, Abdelaziz Banani

**Affiliations:** 1 Service de Gynéco-obstétrique I, CHU Hassan II, Fès, Maroc

**Keywords:** Grossesse hétérotopique, cœlioscopie, pronostic de la grossesse, Heterotopic pregnancy, coelioscopy, pregnancy prognosis

## Abstract

La grossesse hétérotopique est définie par la coexistence d'une grossesse intrautérine (GIU) et d'une grossesse extra-utérine (GEU), quelle que soit sa localisation. C'est une forme de la grossesse gémellaire dizygote bi-ovulaire, sa survenue sur un cycle spontané est rare. C'est une pathologie rare et grave qui peut parfois mettre en jeu le pronostic vital maternel. Nous rapportons deux observations de patientes qui ont bénéficié d'une prise en charge pour une grossesse hétérotopique au Service de Gynécologie-obstétrique I, CHU Hassan II de Fès, sur une période d'une année. Nous avons rapporté les données cliniques, échographiques ainsi que la prise en charge thérapeutique de cette pathologie. La douleur pelvienne était le motif de consultation principal. Le diagnostic de grossesse hétérotopique a été suspecté à l'échographie pour les deux cas. Le traitement réalisé était un traitement conservateur par voie coelioscopique pour le premier et une salpingectomie par mini-laparotomie transverse pour le deuxième. L'évolution de la GIU était favorable dans les deux cas.

## Abstract (en)

Heterotopic pregnancy is defined as the coexistence of intrauterine pregnancy (IP) and ectopic pregnancy (EP), regardless of its location. It is a type of bi-ovular dizygotic twin pregnancy, its occurrence in a spontaneous cycle is rare. This is a rare and serious pathology which may compromise maternal prognosis. We report two cases of heterotopic pregnancy treated in the Department of Gynecology and Obstetrics I at the University Hospital Hassan II of Fez, over a one-year period. Clinical, ecographic and therapeutic data of this pathology were reported. Pelvic pain was the main reason for consultation. The diagnosis of heterotopic pregnancy was suspected on ultrasound in both cases. The first patient underwent conservative treatment by laparoscopy while the second patient underwent salpingectomy by mini-transverse laparotomy. IP outcome was favorable both cases.

## Introduction

La grossesse hétérotopique ou diplotique est définie par la coexistence d'une grossesse intra-utérine (GIU) et d'une grossesse extra-utérine (GEU) quelle que soit sa localisation, chez la même patiente. C'est une pathologie souvent méconnue qui pose un problème diagnostique et qui peut entraver le pronostic vital de la femme. Nous en rapportons deux cas à travers lesquels nous décrirons les modalités diagnostiques, la prise en charge de la GEU et l'évolution de la grossesse.

## Patient et observation

### Observation 1

**Figure 1:**
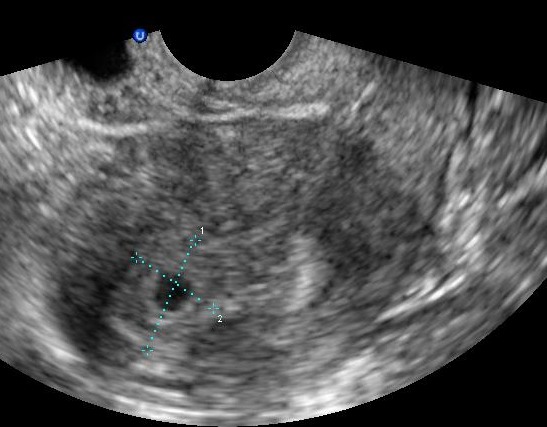
Echographie de la grossesse intra-utérine

**Figure 2:**
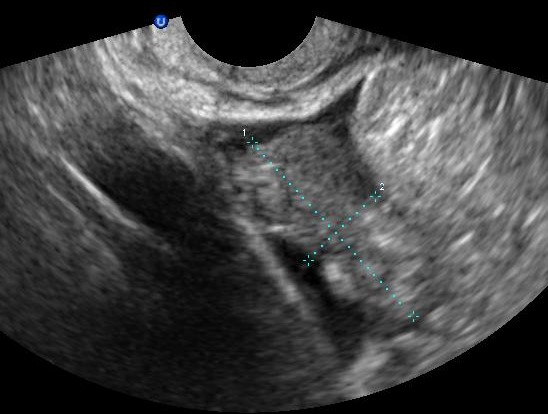
Image échogène hétérogène en faveur d'un hématosalpinx

**Figure 3:**
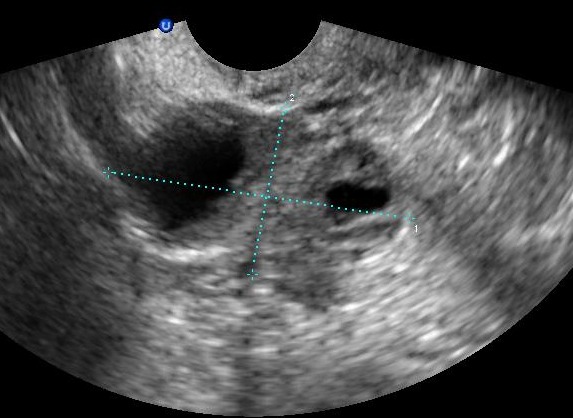
Echographie des deux corps jaunes

**Figure 4:**
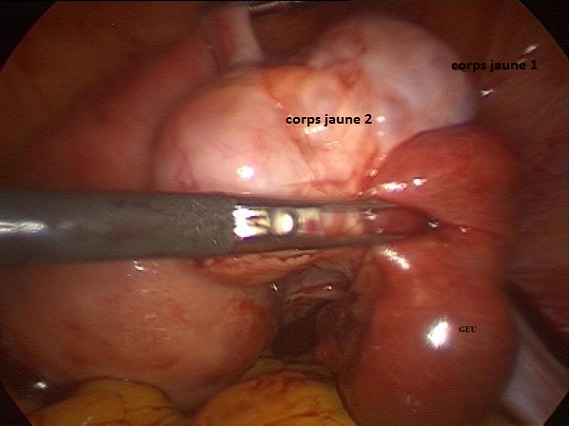
Image coelioscopique montrant l'hématosalpinx droit avec les 2 corps jaunes

**Figure 5:**
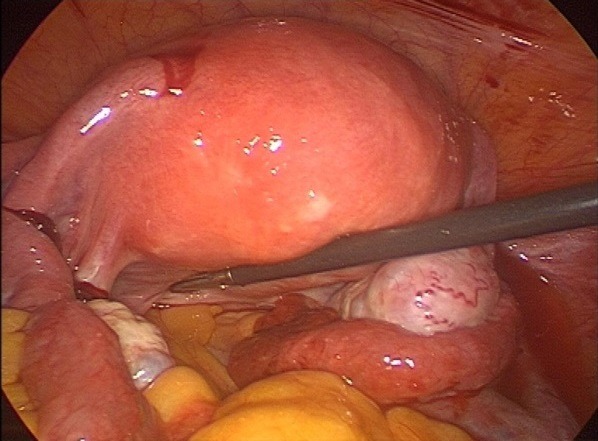
Image coelioscopique après évacuation de la GEU

Mme S.K âgée de 31 ans, mère de 2 enfants et porteuse d'un utérus cicatriciel. La patiente est sous contraception orale depuis 12 mois. Elle consulte aux urgences pour une douleur pelvienne aigue associée à des métrorragies de faibles abondances avec notion de retard de règles. À l'admission, les constantes hémodynamiques étaientstables. L'abdomen était souple avec une légère sensibilitéde la fosse iliaque droite. Au spéculum, col gravide avec présence d'un saignement minime provenant de l'endocol. Au toucher vaginal, l'utérus était de taille subnormale, le CDS latéral droit était douloureux sans masse palpable. À la biologie, le taux d'hémoglobine était à 12g/dL, le taux de BHCG à 3187 UI /ml. À l'échographie endovaginale, présence d'un sac gestationnel intra-utérinsans embryon, avec présence d'une double image ovarienne droite anéchogène évoquant deux corps jaunes associée à une image échogène hétérogène latéro-ovarienne de 4x3cm faisant évoqué une GEU ainsi qu'un épanchement de faible abondance (Figure 1, Figure 2, Figure 3). Une cœlioscopie diagnostique a été réalisée sans canulation utérine. L'exploration a objectivé un hémopéritoine de 50mL, une GEU droite au niveau du pavillon de 4cm au stade d'hématosalpinx. L'utérus était légèrement augmenté de taille, d'aspect gravide (Figure 4). Une expression tubaire a été réalisée évacuant tout le tissu trophoblastique (Figure 5). Les suites opératoires étaient simples. Un contrôle échographique après 2 semaines a montré une évolution normale de la grossesse.

### Observation 2

Mme H.M est âgée de 21ans, primigeste avec notion de cycle irrégulier. Dans ses antécédents, on note une tuberculose pulmonaire traitée et déclarée guérieil y a un an. Elle consulte aux urgences pour des algies pelviennes aigues associées à des métrorragies sur une aménorrhée de 2 mois. L'examen clinique trouve une patiente consciente stable sur le plan hémodynamique et respiratoire, avec présence de métrorragies minimes provenant de l'endocol et une sensibilité latéro-utérine gauche. L'échographie pelvienne est en faveur d'un sac gestationnel intra-utérin avec vésicule vitelline avec à une image échogène hétérogène latéro-utérine gauche de 3x2cm faisant évoquer un hématosalpinx, associée à un épanchement intrapéritonéal de moyen abondance. La patiente a bénéficié d'une mini laparotomie transverse objectivant un hématosalpinx ampullaire gauche de 3cm, nous avons pratiqué une salpingotomie avec évacuation de la grossesse. Evolution normale de la grossesse jusqu'à 36 semaines ou un déclenchement a été indiqué pour oligoamnios avec accouchement par voie basse sans particulier.

## Discussion

La grossesse hétérotopique est l'association d'une GIU et d'une GEU chez la même patiente. Le premier cas a été rapporté par Duvernet en 1708 au cours d'une autopsie [1]. Sur le plan épidémiologique, la fréquence de la grossesse hétérotopique a augmenté depuis l'avènement de la procréation médicalement assistée, et également en raison de la recrudescence des infections génitales hautes, des chirurgies tubaires..., néanmoins sa survenue sur une grossesse spontanée reste rare, variant entre 1/30000 à 2/10000 [2,3]. Les facteurs de risque de la grossesse hétérotopique spontanée sont ceux de la GEU. L'infection génitale est le principal facteur de risque, surtout les infections subaiguës ou chroniques par le Chlamydia qui passent en général inaperçu [4]. Les antécédents de GEU ou l'obstruction tubaire représentent également des facteurs de risques important pour la survenue de GH. Sur le plan physiopathologique, la grossesse combinée peut résulter d'une fécondation simultanée (différence de vitesse de migration de deux ovules fécondés) ou d'une fécondation différée (fécondation de deux ovules produits à un court intervalle au cours d'un même cycle par deux spermatozoïdes provenant de deux coïts successifs) [3,5]. Les circonstances de découverte d'une grossesse hétérotopique sont variables. Le diagnostic est facile quand les signes de la GEU sont au premier plan, la symptomatologie clinique est alors dominée par latriade classique de la GEU: aménorrhée, métrorragies dans 50% des cas, douleurs pelviennes dans 82,7 à 90% des cas. Un collapsus peut également se voir chez 13 à 45% des patientes [5]. L'association de cette triade à une augmentation du volume utérin est fortement évocatrice de grossesse hétérotopique. Le diagnostic est plus difficile si le tableau clinique est celui d'une grossesse intra-utérine. La symptomatologie clinique est souvent rattachée à une menace d'avortement ou à un avortement en cours, le diagnostic de grossesse hétérotopique n'est posé qu'à l'apparition des signes d'un hémopéritoine secondaire à une rupture de la GEU [3] associée ou non à un état de choc maternel [6], souvent mortel. Enfin la grossesse hétérotopique peut être totalement asymptomatique découverte de façon fortuite lors d'un examen échographique [7].

L'échographie pelvienne est le principal examen paraclinique qui permet de poserle diagnostic d'une grossesse hétérotopique [7]. L'examen des annexes doit être systématique après la mise en évidence de la GIU, surtout chez les patientes ayant des facteurs de risque de faire une GEU [6]. L'échographie permet de poser le diagnostic de préciser l'âge de la grossesse, le siège de la GEU, l'évolutivité de la GIU, et de rechercher d'éventuelles complications [2]. Les éléments échographiques qui permettent d'affirmer une grossesse hétérotopique sont la présence d'un sac gestationnel intra-utérin contenant un embryon associé à une masse latéro-utérine échogène hétérogène, ou complètement anéchogène entourée d'un halo trophoblastique échogène, contenant parfois un embryon, associée ou non à un épanchement dans le cul de sac de Douglas [2]. Le principe de la prise en charge de la grossesse hétérotopique consiste à intervenir sur la GEU tout en essayant de préserver la GIU. La cœlioscopie exploratrice doit être effectuée en première intention en cas de doute diagnostic pour le confirmer et pour permettre de préserver au maximum le pronostic de la GIU [8]. Le traitement doit être conservateur tant que c'est possible. En cas d'hémorragie intra-abdominale ou d'état de choc, une laparotomie est préférable [6]. Le traitement médical peut être une alternative si la GIU n'est pas évolutive. Enfin, le pronostic de la GIU dépend surtout de la précocité du diagnostic [9]. 30 à 75% des GIU évoluent à terme après traitement de la GEU [10, 11]. Quel que soit l'abord chirurgical, ça ne semble pas perturber le développement de la GIU, à condition que la manipulation de l'utérus soit minimale et l'anesthésie de courte durée.

## Conclusion

La fréquence des grossesses hétérotopiques est en nette ascension à cause de l'augmentation des facteurs favorisants la GEU. Le diagnostic de grossesse hétérotopique ne doit pas être exclu par la découverte d'une GIU sur un cycle spontané. Elle doit être suspectée devant toute apparition de douleur abdominale, la présence d'une masse latéro-utérine avec ou sans écho embryonnaire visible et/ou présence d'épanchement intra-péritonéal au premier trimestre de la grossesse. La cœlioscopie est à la fois diagnostique et surtout thérapeutique. Elle permet le traitement de la GEU tout en restant conservateur pour la GIU.

## Conflits d’intérêts

Les auteurs ne déclarent aucun conflit d'intérêts.
